# The Role of Intrinsic Defects in the Structural Transition of Superconducting La_3_Ni_2_O_7_: A Many-Body DMC Prediction

**DOI:** 10.1021/acs.chemmater.6c01607

**Published:** 2026-09-04

**Authors:** Abdul Ghaffar, Kayahan Saritas, Fernando A. Reboredo

**Affiliations:** Materials Science and Technology Division, 6146 Oak Ridge National Laboratory, Oak Ridge, Tennessee 37831, United States

## Abstract

The pressure-induced structural phase transitions of the superconducting Ruddlesden–Popper nickelate La_3_Ni_2_O_7_ remain under active debate. While the *Amam* phase is generally identified as the non-superconducting ambient-pressure structure, several higher-symmetry phases have been proposed to emerge under compression; these phases would potentially host superconductivity. Adding to this complexity, experimental and theoretical studies consistently report oxygen vacancies in La_3_Ni_2_O_7_, raising questions about the impact of oxygen vacancies and other intrinsic defects on the material’s electronic and superconducting properties. Although vacancies are known to strongly affect the electronic properties of oxides in general, their influence on the relative stability of the high-pressure phases of La_3_Ni_2_O_7_ has not been examined. To address this gap, we employ many-body diffusion Monte Carlo to investigate the role of oxygen vacancy defects in La_3_Ni_2_O_7_. We apply this approach across the structural phases *Amam*, *Fmmm*, *I*4/*mmm*, and *Cmmm* at T = 0 to obtain a comprehensive understanding of their defect energetics. Our results show that departures from oxygen stoichiometry significantly alter the stability of the high-pressure phases. In particular, the oxygen vacancy stabilizes the experimentally observed *Fmmm* phase at higher pressures compared to the stoichiometric phases. Our findings indicate that vacancy formation under pressure may act as a driving mechanism for the phase transition, providing new insight into the stabilization pathways of La_3_Ni_2_O_7_ and their connection to possible superconducting behavior.

## Graphical abstract

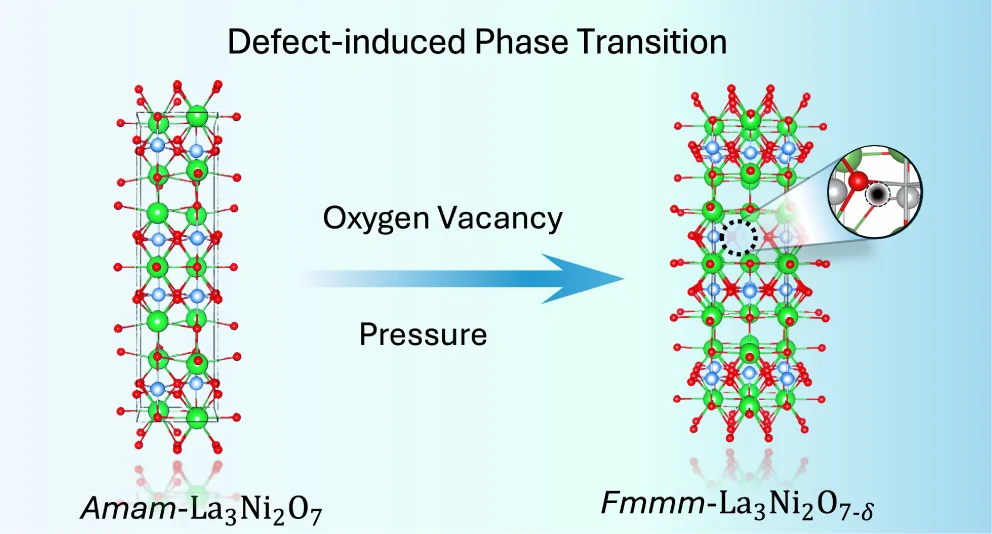

## 1 Introduction

The demand for highly accurate theoretical predictions is intensified for materials at extreme conditions because standard experimental characterization techniques become impractical. This situation arises, for example, during high-pressure experiments, in particular, during the phase transitions associated with superconductivity in La_3_Ni_2_O_7_.

Ruddlesden–Popper nickelates, with the general formula La_n+1_Ni_n_O_3n+1_, have emerged as fertile ground for exploring correlated-electron phenomena, owing to their layered structure, tunable bandwidth, and multivalent nickel states. Optical studies of the series reveal that as *n* increases from bilayer (*n* = 2) to trilayer (*n* = 3) to infinite-layer (*n* = ∞), the ratio of experimental to band-theory kinetic energy, *K*_exp_/*K*_band_, increases,^1^ suggesting strongest deviation from the band-theory at the *n* = 2 limit in this series. This deviation suggests tunable electronic correlations in nickelates. Notably, the *n* = 2 member, La_3_Ni_2_O_7_, lies on the verge of a Mott-insulating regime,^1^ underscoring its delicate balance between itinerancy and localization.^1,2^ La_3_Ni_2_O_7_ is particularly compelling because its electronic and lattice degrees of freedom can be dramatically reshaped by pressure, cation/anion stoichiometry, and spin order.^3–5^ Among these factors, oxygen nonstoichiometry plays an significant role: oxygen vacancies directly modify local coordination, crystal-field splitting, and nickel valence.^6^ Oxygen vacancies couple the lattice, charge, and spin degrees of freedom, potentially tuning the related emergent properties in La_3_Ni_2_O_7_.^7^ Such tunability could tip the balance among competing phases.

Recent work on La_3_Ni_2_O_7_ has rapidly broadened the structural and defect landscape, but key gaps remain to be understood at the intersection of pressure, vacancies, and accurate energetics.^7–10^ High-pressure superconductivity near 80 K has been reported, coincident with an orthorhombic *Fmmm* phase and competing structural characterization under compression, including evidence for a tetragonal *I*4/*mmm* phase around ∼19 GPa at low temperature, underscoring phase competition under pressure, which is yet to be addressed systematically.^7,11,12^ More recent developments reveal additional polymorphs and stacking variants at ambient conditions (e.g., a long-range ordered “1313” phase in *Cmmm*), signaling that multiple crystallographic motifs can be stabilized in closely related thermodynamic windows.^13–15^ Oxygen vacancies are commonly present in bulk crystals and in thin films, with compositions often reported as O_7−δ_. Studies show that *δ* varies across a measurable range^6^ and is highly sensitive to factors such as annealing conditions and epitaxial strain.^7,16,17^ These observations highlight the challenges of reproducibility and indicate that vacancy chemistry may play a central role in governing transport properties and phase stability.^8,18^

However, the problem of phase stability of La_3_Ni_2_O_7_, its magnetic phase diagram, and defect energetics is a significant challenge for *ab initio* theories that must account for extreme pressures while accurately accounting for correlations without adjustable parameters. Accurate theoretical calculations of vacancy formation enthalpies and their impact on phase stability under pressure remains challenging in systems dominated by strong correlation. However, many-body diffusion Monte Carlo (DMC)^19^ has delivered benchmark-level defect formation energies in complex oxides (e.g., oxygen vacancies in ZnO and perovskites such as LaFeO_3_ and SrFeO_3_). DMC calculations provide excellent agreement with the equation of state of multiple correlated materials under pressure.^20–23^ Additionally, DMC has been successfully applied to predict the structural and electronic properties of several other metal oxides, including manganese oxides (e.g., MnO, MnO_2_, LaMnO_3_)^23^ LiNiO_2_ ^24,25^ and NiO.^26^ However, the influence of pressure on the formation energy of defects in correlated materials has not been studied with DMC. Moreover, DMC analyses of vacancy-driven stabilization of material phases under pressure are absent.

This paper details the many-body DMC and complementary density functional theory (DFT) calculations, within PBE+*U* approximation^27,28^ , employed to systematically investigate oxygen vacancies across four reported structural phases of La_3_Ni_2_O_7_; *Amam*, *Cmmm*, *Fmmm*, and *I*4/*mmm*. We resolved their magnetic ground states, calculated the site-dependent vacancy formation energy, and compared the pressure-driven energy differences of both stoichiometric and defect-containing structures. We uncovered a pronounced dependence between oxygen stoichiometry and phase stability. Our results reveal that vacancies can decide the energy ordering of the high-pressure phases. Our results reproduce the experimentally observed emergence of the *Fmmm* phase at elevated pressures (where superconductivity is observed) only when oxygen vacancies are present. These findings position oxygen vacancies as an essential component to elucidate the structural evolution of La_3_Ni_2_O_7_ and provide a quantitative framework for interpreting ongoing high-pressure experiments in this important nickelate superconductor system.

## 2 Methodology

### 2.1 General Approach

A comparative analysis of DFT (using the Perdew–Burke–Ernzerhof [PBE] generalized gradient approximation [GGA] functional) and DMC results was conducted to draw systematic conclusions. This comparison clarifies the roles of oxygen vacancies and magnetic ordering in determining phase stability under elevated pressures. To perform this analysis, we employed a multistep computational workflow using first DFT and then DMC calculations.

First, the crystal structures of the relevant phases were transformed into supercells containing four formula units, ensuring the use of the most physically isotropic configurations with the largest Wigner radii. This choice facilitates finite-size error cancellation in DMC calculations. To determine the lowest-energy magnetic ordering for each phase, a comprehensive analysis of several magnetic configurations was performed using a PBE+*U*-based DFT approach. In this approach, the parameter *U* acts as an effective on-site Coulomb interaction applied to the localized states. For consistency and to capture the magnetic behavior at high pressure, these calculations were performed at 20 GPa, where the high-pressure phases compete.^29^ This magnetic ground state ordering on nickel atoms was then used as the starting point for subsequent oxygen vacancy formation energy calculations within the same PBE+*U* framework. Similarly, we calculated the magnetic ground states for each phase using DMC. Next, we systematically identified all inequivalent oxygen lattice sites within each phase to enable targeted vacancy formation studies.

Next, trial wave functions resulting from PBE+*U* calculations were employed in DMC to enforce the fixed-phase approximation. DMC calculations were carried out to assess both the magnetic energy ordering and the energetics of oxygen vacancies across different crystallographic phases for different pressures.

To model oxygen vacancy defect systems, we introduced a single oxygen vacancy into four-formula-unit (f.u.) supercells and evaluated all symmetrically inequivalent vacancy sites across different structural phases. Recent multiscale electron ptychography (MEP) measurements^6^ have revealed nanoscale regions with varying oxygen deficiency in La_3_Ni_2_O_7_. In particular, these measurements show that La_3_Ni_2_O_7−δ_ compositions exhibit *δ* values ranging from 0.04 ± 0.11 to 0.34 ± 0.22. Here, the parameter *δ* is used to quantify the oxygen deficiency. Our theoretical model, which introduces a single oxygen vacancy in periodic boundary conditions (a 4-f.u. supercell with 28 oxygen atoms), corresponds to *δ* = 0.25. This defect concentration is well within the experimentally observed range.

### 2.2 Technical Details

Geometry relaxations were performed using DFT calculations in the Vienna *Ab initio* Simulation Package (VASP)^30,31^ employing the projector augmented-wave (PAW) method^32^ and the PBE GGA^27^ for the exchange-correlation functional. To account for strong on-site Coulomb interactions on nickel 3*d* orbitals, we applied a Hubbard *U* correction^28^ with *U* = 3.5 eV, consistent with previous studies^7,9,33–35^ on La_3_Ni_2_O_7_ systems. Our test of the DFT oxygen-vacancy formation energy with a different *U* value yields relatively small difference (see Table S9 of the Supporting Information), indicating that the defect energetics relevant to the PBE+*U* comparison are robust against moderate changes in *U*.

All crystal phases were structurally optimized with PBE+*U* in a spin-polarized setup under external pressures. A plane-wave energy cutoff of 600 eV was used throughout to ensure convergence of the total energy. Given the metallic nature of the La_3_Ni_2_O_7_ phases, Gaussian smearing with a width of 0.05 eV was employed for Brillouin zone integrations. The reciprocal space was sampled using a Γ-centered Monkhorst–Pack *k*-point mesh with a density of 0.2 Å^–1^, defined in units of 2*π*/*L*_*i*_, where *L*_*i*_ denotes the lattice constant along the *i*th crystallographic direction. Structural optimizations were performed until the total energy and atomic forces converged below thresholds of 1 × 10^–5^ eV and 1 × 10^–2^ eV/Å, respectively.

DMC simulations were performed using the QMCPACK package.^36^ Trial wavefunctions for the DMC calculations were generated using the Quantum ESPRESSO *ab initio* package^37^ employing the PBE-GGA exchange-correlation functional.^27^ To obtain a good-quality trial wavefunction, a plane-wave basis set with a kinetic energy cutoff of 500 Ry and a charge-density cutoff of 2000 Ry was used for the convergence. The pseudopotentials used for generating the trial wavefunctions have hard nuclear cores. These pseudopotentials are optimized for accurate treatment of correlation effects and typically require very high plane-wave cutoffs. We employed correlation-consistent effective core potentials for nickel^38^ and oxygen^39^ atoms, and a norm-conserving pseudopotential generated using the OPIUM package^40^ was used for the lanthanum atom. Brillouin zone integrations were carried out using Gaussian smearing with a width of 0.001 Ry. The self-consistency threshold for the PBE+*U* calculations used to generate the trial wavefunction was set to 1 × 10^–7^ Ry. Using *U* = 3.5 eV to generate the trial wavefunctions used in our DMC calculations is consistent with previous DMC benchmarks for related nickel oxides, where DMC energies remain largely unchanged for a *U* (Ni) values ranging from 3 to 4 eV.^24–26,41^ All other DFT parameters at this stage were kept consistent with those used in the VASP calculations.

The DMC simulations were carried out using the Nexus automation utility^42^ within QMCPACK^36^ starting from the trial wavefunction generation stage. The nonlocal components of the pseudopotentials were treated using the revised T-moves approximation^43^ within DMC, ensuring improved stability and accuracy in the presence of nonlocal pseudopotentials. To incorporate electron correlation effects beyond the mean-field level, Slater–Jastrow trial wavefunctions were employed, with the Jastrow factors optimized via variational Monte Carlo (VMC) using Umrigar’s linear method.^44^ An imaginary time step of 0.01 Ha^–1^ was used for all DMC runs. A representative time-step test for the *Fmmm* phase, performed at 0.02 Ha^–1^, yielded a relative energy difference (0.003 Ha) within the error bar of the 0.01 Ha^–1^ result (SI, Table S10), confirming that the reported energetic trends are insensitive to this choice within statistical uncertainty. To mitigate finite-size effects, twist-averaged boundary conditions were applied using uniform Γ-centered grids across all supercells.^45^ Specifically, the twist-grid sizes were 4 × 3 × 4 for the *Amam* phase, 6 × 6 × 2 for the *Cmmm* phase, and 4 × 4 × 3 for both the *Fmmm* and *I*4/*mmm* phases.

### 2.3 Theoretical Background on DMC

The DMC method^46,47^ implemented within the quantum Monte Carlo framework, with real-space fixed-node approximation, offers a highly accurate approach for solving the many-body Schrödinger equation. DMC stochastically projects out the ground-state component of a trial wavefunction *Ψ*_*T*_(**R**) by evolving it in imaginary time *τ* according to the time-dependent Schrödinger equation:

1 $$\begin{array}{ll} \frac{\partial f\left(R,\tau \right)}{\partial \tau } & =\frac{\partial }{\partial \tau }\left[\Psi_{T}\left(R\right)\Psi \left(R,\tau \right)\right]\\ =\Psi_{T}\left(R\right)\frac{\partial \Psi \left(R,\tau \right)}{\partial \tau },\\ -\Psi_{T}\left(R\right)\frac{\partial \Psi \left(R,\tau \right)}{\partial \tau } & =\Psi_{T}\left(R\right)\left(H-E_{T}\right)\Psi \left(R,\tau \right)\\ \Rightarrow \frac{\partial f\left(R,\tau \right)}{\partial \tau } & =-\Psi_{T}\left(R\right)\left(H-E_{T}\right)\Psi \left(R,\tau \right)\\ \mathrm{with}\;f\left(R,\tau \right) & \equiv \Psi_{T}\left(R\right)\Psi \left(R,\tau \right) \end{array}$$

where **R** denotes the many-electron configuration, H is the many-body Hamiltonian, and *E*_*T*_ is a reference energy. eq 1 can be rewritten as a diffusion process with branching.^46,47^

2 $$\frac{\partial f\left(R,\tau \right)}{\partial \tau }=\frac{1}{2}\nabla^{2}f\left(R,\tau \right)-\nabla \cdot \left[F\left(R\right)f\left(R,\tau \right)\right]-\left(E_{L}\left(R\right)-E_{T}\right)f\left(R,\tau \right)$$

with

$$F\left(R\right)=\nabla \ln|\Psi_{T}\left(R\right)|,,E_{L}\left(R\right)=\frac{H\Psi_{T}\left(R\right)}{\Psi_{T}\left(R\right)}$$

The importance-sampled distribution *f*(**R**,*τ*) = Ψ_*T*_(**R**)­Ψ­(**R**,*τ*) evolves according to a Fokker–Planck-type equation^46,47^ containing diffusion, drift, and branching contributions. The Laplacian term, $\frac{1}{2}\nabla^{2}f$ , represents the bare quantum diffusion inherited from the kinetic operator. The drift arises from the gradient of the logarithm of the trial wavefunction, **F**(**R**), which biases the random walk toward regions where Ψ_*T*_ is large, causing the importance sampling and enforcing the fixed-node (phase) approximation. The final term, −(*E*_*L*_(**R**)–*E*_*T*_)*f*, weights the distribution by the difference between the local energy *E*_*L*_(**R**) and the reference energy *E*_*T*_, generating the branching process that drives Ψ­(**R**,*τ*) toward the fixed-node (phase) ground state.

In the long-time limit, the quantity *f* converges to the product *f* = Ψ_*T*_(**R**) Ψ_0_(**R**), where Ψ_0_(**R**) is the fixed-node (phase) ground state. To mitigate the Fermion sign (phase) problem, DMC employs the fixed-node (phase) approximation. The term Ψ_0_(**R**) is constrained to have the same node (phase) as the trial wavefunction Ψ_*T*_, introducing a positive error dependent on the quality of the trial wavefunction nodes (phase). The total energy in DMC is obtained via the mixed estimator:

3 $$E_{\mathrm{DMC}}=\frac{\int dRf\left(R\right)E_{L}\left(R\right)}{\int dRf\left(R\right)}$$

which becomes exact if the nodes (phase) of Ψ_*T*_ coincide with the ones of true ground-state wavefunction. In practice, Slater–Jastrow forms are employed for Ψ_*T*_, where the Jastrow factor partially includes electron–electron and electron-ion correlations beyond the reach of single-particle mean-field approximations such as conventional DFT. The Jastrow parameter is usually optimized by using VMC before a DMC run. This procedure enables DMC to achieve many-body benchmark-level accuracy in predicting ground-state properties. Many-body DMC offers a systematically more accurate treatment of electronic correlations than DFT approaches, making it a more suitable tool for elucidating the physics of strongly correlated nickelates such as La_3_Ni_2_O_7_.

### 2.4 Evaluation of Defect Formation under Pressure

At zero temperature, enthalpy determines the stability of two compounds with identical composition via *H* = *U* + *PV*, where *U* is the internal energy, *P* is pressure, and *V* is volume. At ambient pressures (still at 0 K) the *PV* term is typically on the order of 10^–5^ eV/atom; therefore, only the internal energy can be used to determine the stability. The internal energy of a system is the central observable for *ab initio* theory, such as DFT and DMC. Although forces and the stress tensors are often readily available in most DFT codes to calculate the *PV* contribution, accessing these quantities is challenging in DMC, especially for extended systems with low or broken structural symmetries.^48,49^ We performed structural optimizations of La_3_Ni_2_O_7_ and La_3_Ni_2_O_7−δ_ at external pressures ranging from 0 to 20 GPa using PBE+*U* implemented within the VASP code. These structures from PBE+*U*, thus the corresponding pressures and the volumes, are subsequently used in DMC calculations and to determine the enthalpy in DMC. Therefore, the enthalpy in DMC was explicitly evaluated as follows:

4 $$H_{\mathrm{DMC}}=E_{\mathrm{DMC}}+P\cdot V$$

where *E*_DMC_ denotes the DMC internal energy, and *V* is the volume of the supercell previously optimized using PBE+*U* at the target external pressure *P*.

We computed the formation energy (FE_VO_) of a neutral single oxygen vacancy under pressure using the following expression:

5 $${\mathrm{FE}}_{\mathrm{VO}}=H_{\mathrm{defect}}-H_{\mathrm{bulk}}+\mu_{O}$$

Here, *H*_defect_ and *H*_bulk_ are the enthalpies of the defective and bulk supercells, respectively. The reference oxygen chemical potential *μ*_O_ is taken as one-half of the energy of an isolated O_2_ molecule. The resulting oxygen vacancy formation energy should be regarded as an upper bound because the oxygen chemical potential in solids cannot exceed that of an isolated O_2_ molecule. The actual thermodynamic cost of creating oxygen vacancies could be lower under realistic conditions. We use this formulation solely to ensure a consistent comparison among the different oxygen vacancy configurations with the same reference. This is sufficient for this study because our focus is on comparison of systems with the same number of vacancies where the chemical potential cancels out. We do not aim to report absolute formation energies for individual configurations.

## 3 Results

### 3.1 Crystal Phases of La_3_Ni_2_O_7_

The phase diagram of La_3_Ni_2_O_7_ reveals the presence of multiple competing structural phases, reflecting the intricate coupling among lattice, electronic, and magnetic degrees of freedom. Among these, the phase with space-group symmetry *Amam* is widely recognized as the ambient-pressure ground state. By contrast, three additional phases—*Fmmm*, *I*4/*mmm*, and *Cmmm*—have been claimed to exist under applied pressure.^13–15,29,50,51^ This suggests the presence of pressure-induced structural transitions that may significantly influence the material’s electronic properties. These phases differ mainly in the structure and stacking sequence of NiO_6_ octahedra. The low-pressure, non-superconducting *Amam* phase has Ni–O–Ni angle of 168° along the apical direction. Increasing pressure stabilizes higher-symmetry phases (*Fmmm* and *I*4/*mmm*) that approach this angle toward 180°.^18,52^ In the *Cmmm* phase, the Ni–O–Ni bond angle is consistently 180° across ambient and elevated pressures.^14,53^ The *Amam*, *Fmmm*, and *I*4/*mmm* phases exhibit a 2222 stacking of nickel layers, whereas *Cmmm* shows a 1313 stacking sequence. The corresponding atomic structures are shown in Figure 1a.

**Figure 1:**
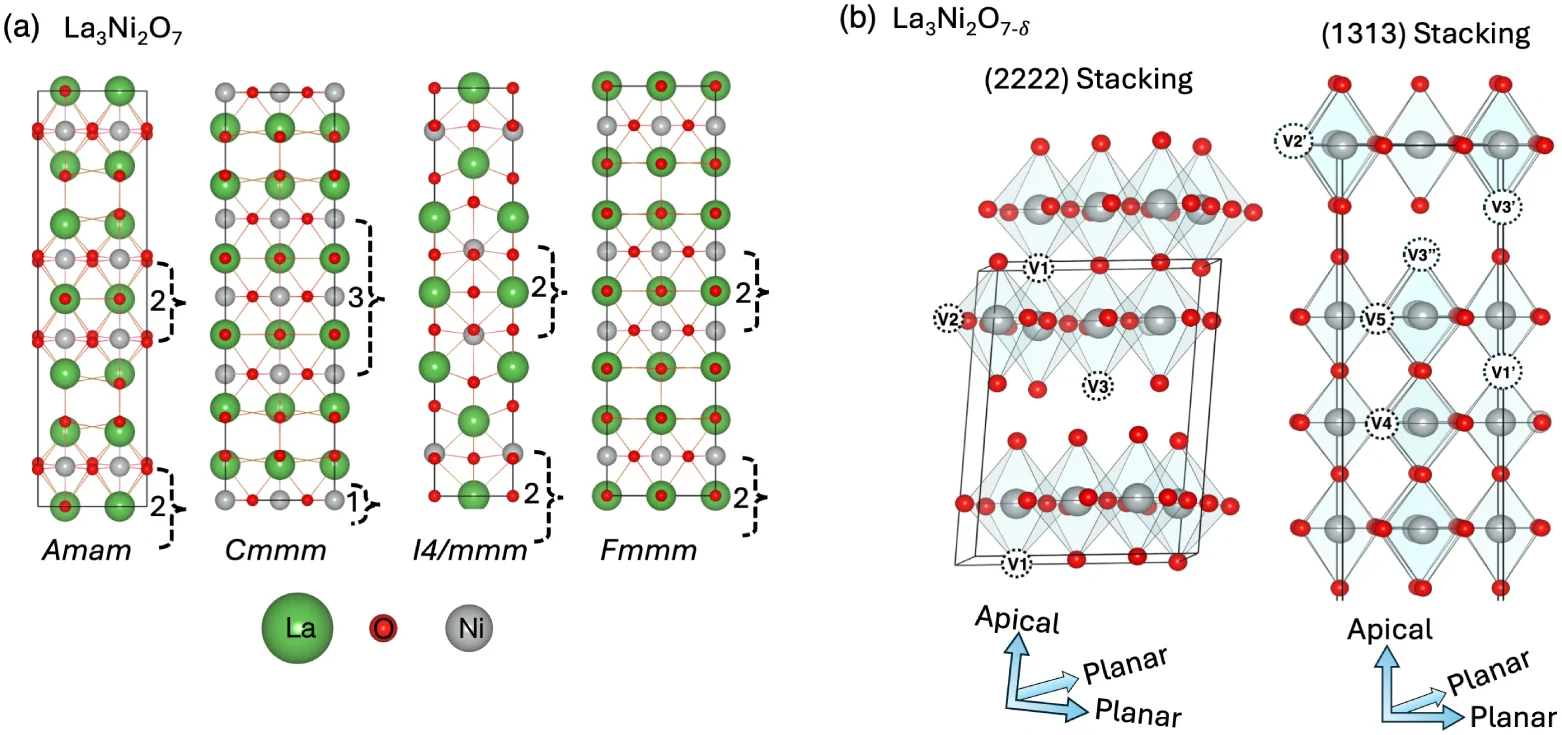
La_3_Ni_2_O_7_ crystal phases and corresponding inequivalent oxygen sites. (a) Crystal structures of each La_3_Ni_2_O_7_ phase are shown in their conventional unit cells. Green, red, and silver spheres represent La, O, and Ni atoms, respectively. The stacking sequences of NiO_6_ octahedra for the *Amam, Cmmm, I*4/*mmm*, and *Fmmm* phases are 2222, 1313, 2222, and 2222, respectively. (b) Visual representation of the NiO_6_ octahedral layers in La_3_Ni_2_O_7_, with inequivalent oxygen vacancy sites indicated by dashed circles. Five symmetrically distinct oxygen sites are shown. The *Amam, Fmmm*, and *I*4/*mmm* phases each contain three unique oxygen positions—inner apical (V1), outer apical (V2), and planar (V3)—while the *Cmmm* phase includes two additional sites: inner planar (V4) and outer planar (V5). Inner apical oxygens lie between corner-sharing NiO_6_ octahedra, outer apical oxygens sit at the interface between the two octahedral layers, and equatorial oxygens reside within the octahedral planes. The inner and outer planar sites arise from the 1313 stacking sequence, corresponding to planar oxygens associated with the central octahedron of three corner-sharing units. La atoms are omitted to highlight the O coordination environment around Ni.

### 3.2 Magnetic Landscape of the La_3_Ni_2_O_7_ Structural Phases

Beyond structural complexity, identifying the magnetic ground state of each phase is essential for understanding the fundamental properties of La_3_Ni_2_O_7_. The coupling between structure and magnetism critically shapes the total-energy landscape and could tune the emergent properties, including superconductivity and charge ordering.^34,50,53^ The underlying magnetic ordering could also affect the formation energy of defects. Accordingly, we first evaluated the relevant magnetic configurations of La_3_NiO_7_ phases using DMC before analyzing the defect energetics of the La_3_Ni_2_O_7_ phases.

We investigated the energies of all the four crystalline phases in four distinct magnetic configurations: one ferromagnetic (FM) and three antiferromagnetic (AFM) arrangements, denoted as A-type, C-type, and G-type. A schematic representation for all magnetic orderings is presented in the (Supporting Information Figure S1); their numerical values are listed in the Supporting Information Tables S1 and S2. The distinction among these AFM configurations arises from differences in spin orientation of nickel atoms within and between the nickel layers. Specifically, the A-type configuration exhibits intralayer FM alignment combined with interlayer AFM coupling. By contrast, C-type and G-type orders both exhibit intralayer AFM alignment but differ in their interlayer coupling: C-type has FM interlayer coupling, whereas G-type has AFM interlayer coupling.

Figure 2a presents the relative energies of various magnetic configurations for the four candidate phases of La_3_Ni_2_O_7_; energies were computed using DMC methods. All energies are referenced to the FM configuration for clarity. As shown, DMC consistently indicates that AFM ordering is energetically favorable across all phases, and FM configurations show significantly higher energies compared to the lowest AFM orderings.

**Figure 2:**
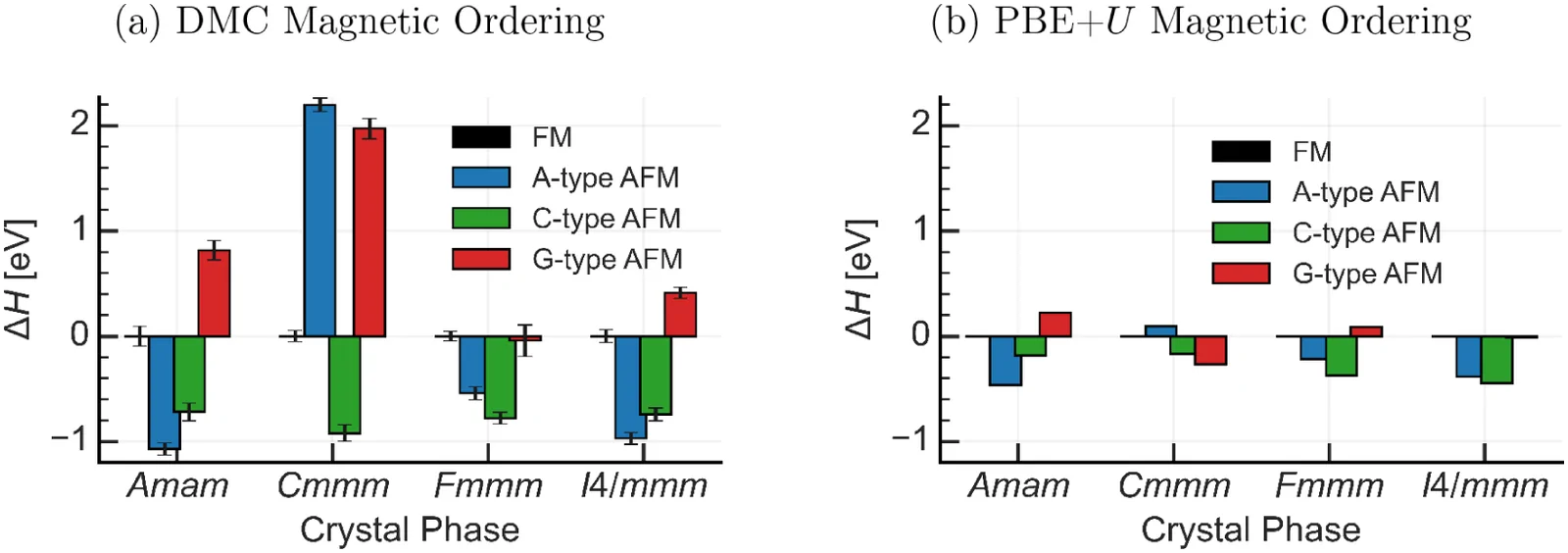
Comparison of magnetic configurations. (a) Bar plot showing DMC magnetic ground state analysis for four La_3_Ni_2_O_7_ phases, each evaluated in four distinct magnetic configurations: one ferromagnetic (FM), and three antiferromagnetic (AFM): A-type, C-type, G-type. Error bars are included on each bar to indicate uncertainty in the DMC energy. (b) PBE+*U* analogue of the same analysis. In both figures, the units for Δ*H* are given in electronvolts (eV) per 4-f.u. cell. Bars are referenced to the energy of the FM configuration corresponding to their respective phases.

Additionally, we performed PBE+*U* calculations for all magnetic configurations, as shown in Figure 2b. These calculations include volume optimization using the PBE+*U* methods. The PBE+*U* results reveal that the *Amam* phase stabilizes in the A-type configuration, *Cmmm* in G-type, and both *I4/mmm* and *Fmmm* in C-type. Notably, the energy differences between the most stable and the second-most stable AFM configurations are relatively small—within 50 meV/f.u. for *Cmmm*, *I4/mmm*, and *Fmmm*—suggesting limited resolution in magnetic ordering at the PBE+*U* level. Although DMC qualitatively agrees with PBE+*U* in favoring AFM over FM states, it exhibits notable discrepancies in identifying the most stable AFM configuration. Specifically, DMC predicts A-type as the ground state for *Amam* and *I4/mmm*, whereas C-type is favored for *Cmmm* and *Fmmm*. A particularly significant deviation is observed in the *Cmmm* phase, where PBE+*U* favors G-type, but DMC identifies C-type as substantially more stable. These differences could indicate the limitations of mean-field approaches like PBE+*U* in capturing the subtle many-body effects governing magnetic interactions in correlated oxides.^54^ The use of high-accuracy many-body DMC calculations for the La_3_Ni_2_O_7_ phases highlights the subtle differences between the two approaches. Furthermore, the larger enthalpy differences of the AFM phases relative to the FM phases in DMC indicate stronger magnetic interactions in DMC than in the PBE+*U*.

La_3_Ni_2_O_7_ has been proposed as an unconventional superconductor, where magnetism plays an important role.^55,56^ Thus, to characterize the magnetic coupling in the structural phases of La_3_Ni_2_O_7_, we extracted the interlayer (*J*_⊥_) and intralayer (*J*_∥_) exchange parameters for each phase (Table 1) from the data in Figure 2. In general, La_3_Ni_2_O_7_ exhibits stronger interlayer exchange coupling (|*J*_⊥_|) than intralayer coupling (|*J*_∥_|), consistent with experiment.^57^ Further discussion and details of the methodology used to compute the *J* parameters are provided in Section 2 of the Supporting Information.

**Table 1:** Comparison of Exchange Parameters, Intralayer Coupling (*J*_∥_) and Interlayer Coupling (*J*_⊥_) from PBE+*U* and DMC, on the Bulk Phases at 20 GPa^a^

	PBE+*U*		DMC	
Phase	*J*_∥_	*J*_⊥_	*J*_∥_	*J*_⊥_
*Amam*	7.02	–24.10	16.24 ± 1.91	–72.39 ± 3.82
*Cmmm*	–14.69	–0.14	–31.89 ± 8.35	141.48 ± 3.85
*I*4/*mmm*	–0.72	–22.40	10.01 ± 1.22	–57.30 ± 2.54
*Fmmm*	–1.04	–22.24	–3.88 ± 2.36	–38.33 ± 6.64

a The units for J are meV per Ni–Ni pair.

### 3.3 Oxygen Vacancy Energetics

Defects can strongly tune the properties of correlated materials such as La_3_Ni_2_O_7_. Among them, oxygen vacancies are the dominant defect in La_3_Ni_2_O_7_ and require careful examination.^6,7,50^ Their influence on the stability of competing phases could be particularly significant. Vacancy formation could serve as a thermodynamic driving force for phase transitions.

From the magnetic ground-state analysis, we selected the lowest-energy magnetic configurations for the oxygen vacancy study. We first calculated the vacancy formation energies at 20 GPa. This pressure was chosen because the high-pressure phases (*Fmmm*, *I*4/*mmm*, and *Cmmm*) are claimed to exist near this regime.^14,29,51,53^ To examine the pressure dependence of vacancy effects, we performed an additional set of DMC calculations at 12 GPa. These results are discussed in Section 3.4 and summarized in Table 2.

**Table 2:** Lowest Oxygen Vacancy Configurations (FE_VO_ in eV Calculated Using 4 f.u. of the Primitive La_3_Ni_2_O_7_ Cell) for the Four Structural Phases of La_3_Ni_2_O_7_, Obtained Using PBE+*U* and DMC Methods at Two Different Pressures: 12 and 20 GPa^a^

	PBE+*U*		DMC	
Configuration	12 GPa	20 GPa	12 GPa	20 GPa
*Amam* (V1)	1.879	1.648	1.597 ± 0.079	1.770 ± 0.099
*Cmmm* (V4)	1.559	1.835	2.842 ± 0.095	2.823 ± 0.108
*I*4/*mmm* (V1)	1.851	1.676	1.675 ± 0.081	2.370 ± 0.143
*Fmmm* (V1)	1.681	1.568	1.576 ± 0.113	0.932 ± 0.107

a Reported uncertainties for DMC values correspond to standard errors.

The *Amam*, *Cmmm*, *I*4/*mmm*, and *Fmmm* phases contain three, six, three, and three inequivalent oxygen sites, respectively, as illustrated in Figure 1b. For the phases with (2222) stacking—*Amam*, *Fmmm*, and *I*4/*mmm*—we label the inner apical, planar, and outer apical sites as V1, V2, and V3. By contrast, the *Cmmm* phase exhibits a (1313) stacking sequence. We label its inner apical, planar, and outer apical sites in the isolated layer as V1′, V2′, and V3′, respectively. The outer apical site in the adjacent three-layer block is labeled as V3″. The prime symbols distinguish these sites from their (2222) counterparts because they are not strictly equivalent. Additionally, the *Cmmm* structure hosts two additional inequivalent V4-like sites (inner planar) and V5 sites (outer planar), which are absent in the other phases.

We evaluated the formation energy of all symmetry-inequivalent single-vacancy sites. First, the vacancy formation energies (FE_VO_) at 20 GPa were calculated, analyzed, and compared with both PBE+*U* and DMC. The FE_VO_ was computed using eq 5.

Figure 3 summarizes the oxygen vacancy formation energies across all structural phases of La_3_Ni_2_O_7_. Figure 3a compares the DMC-calculated FE_VO_ values for all inequivalent oxygen sites at 20 GPa across the four phases. For the (2222) stacking phases—*Amam*, *Fmmm*, and *I*4/*mmm*—the inner apical site (V1) exhibits the lowest formation energy. This observation agrees with recent experimental work.^6^ By contrast, in the (1313) stacked *Cmmm* phase, the most favorable vacancy location is the inner planar site (V4). Among the lowest-energy vacancy sites of each phase, the sequence of increasing formation energy is *Fmmm* < *Amam* < *I*4/*mmm* < *Cmmm*. Also, in general, the hierarchy of FE_VO_ follows the trend inner apical < planar < outer apical.

**Figure 3:**
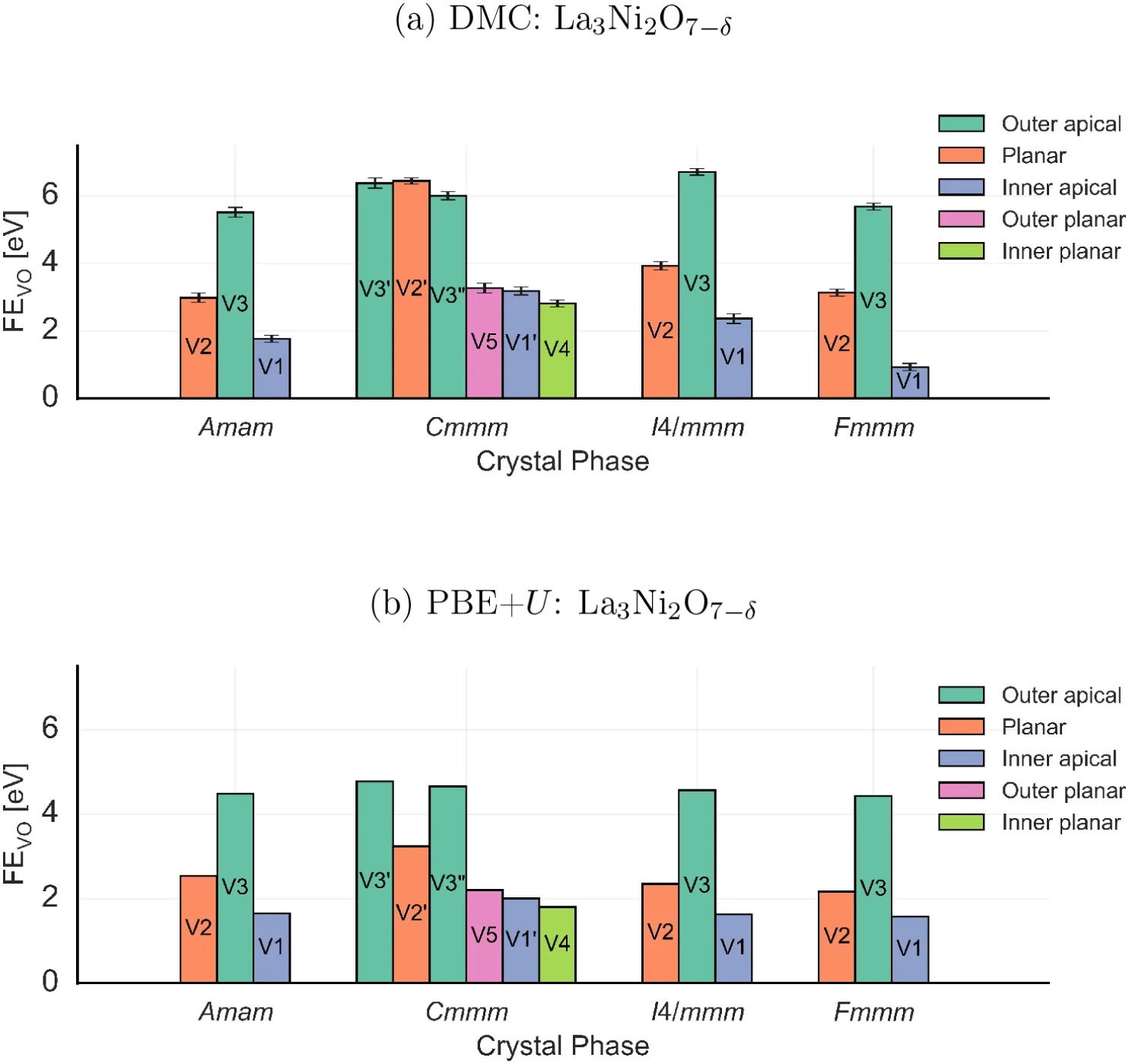
Oxygen vacancy formation energy. (a) Bar plot showing DMC-calculated formation energies for all distinct inequivalent oxygen sites across the four structural phases of La_3_Ni_2_O_7_ at 20 GPa. Error bars are included on each bar to indicate the statistical uncertainty in the DMC formation energies. (b) Corresponding bar plot showing DFT-calculated oxygen vacancy formation energies. The colors of the bars correspond to the physical location of the vacancy sites. Labels shown within the bars correspond to the location of the vacancies as explained in Figure 1.

Figure 3b shows the corresponding PBE+*U* oxygen vacancy formation energies results. PBE+*U* reproduces the same qualitative ordering of FE_VO_. However, the quantitative distinctions as listed in Table 2 are much smaller than in DMC. For example, the difference between the lowest-energy *Amam* and *I*4/*mmm* vacancies is below 10 meV/f.u. in PBE+*U* while in DMC is 150 ± 44 meV/f.u. We note that the difference in PBE+*U* is too small to reliably differentiate and affect the ordering of the phases. By contrast, as depicted in Figure 3 and Table 2, DMC yields clearly different formation energies across all phases. This variation implies that oxygen deficiency could modify the phase stability of the competing high-pressure phases. As a result, the phase transitions in the presence of vacancies may shift as compared to the stoichiometric bulk. This vacancy sensitivity must therefore be considered when analyzing and comparing the experimental and theoretical phase diagrams of La_3_Ni_2_O_7_.

### 3.4 Comparison of Pristine and Vacancy-Containing Phases as a Function of Pressure

Figure 4 presents the comparison of the enthalpies relative to the ambient pressure *A*_*mmm*_ phase for bulk structures and those containing oxygen vacancies as a function of pressure. For the phases with oxygen vacancies, our analysis is limited to the vacancy sites that show the lowest FE_VO_ (Table 2). The rationale for comparing pressure dependence of the enthalpy difference for stoichiometric cases and oxygen-deficient cases is to isolate the role of vacancy defects in the stability ordering of the competing phases under pressure. All enthalpies are relative to the ambient-pressure phase *Amam* to enable evaluation of their influence on the phase transition that drives superconductivity. We note that the transition into phases other than the ambient *Amam* structure occurs at elevated pressures, making this choice of reference appropriate for a clear comparison.

**Figure 4:**
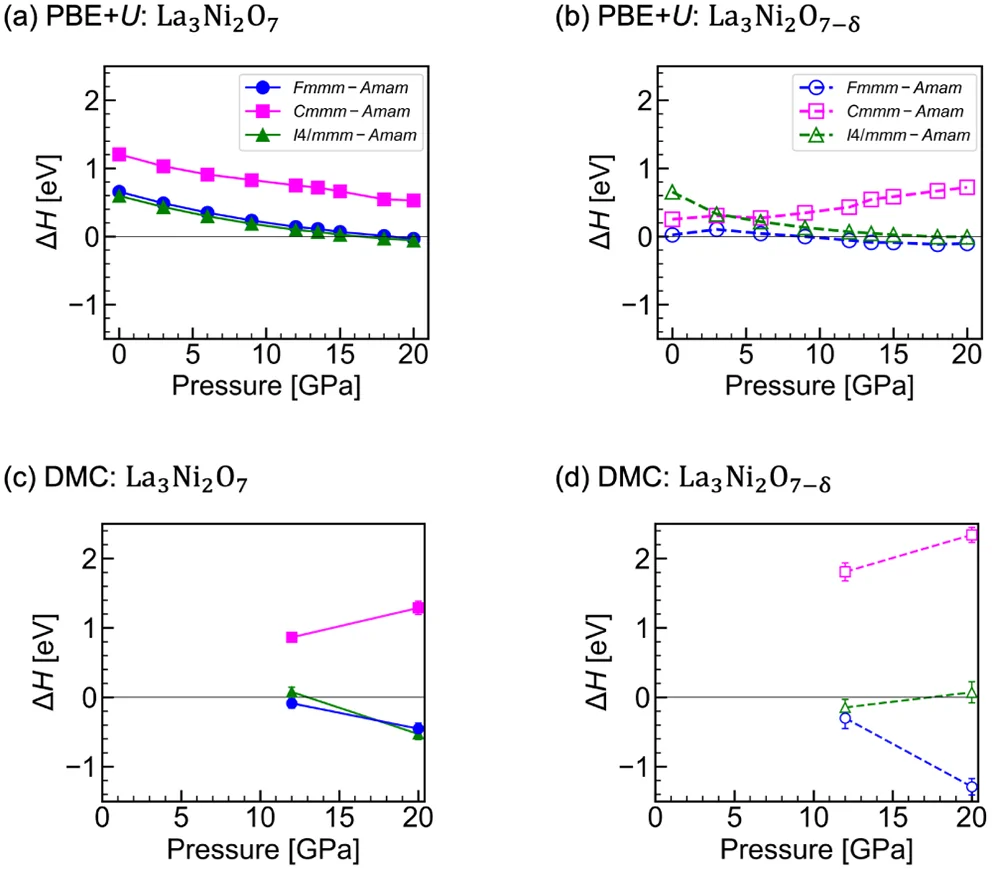
Relative enthalpy of high-pressure phases. Panels (a) and (b) show the pressure-dependent enthalpy of the three high-pressure phases (*Cmmm, I*4/*mmm*, and *Fmmm*) relative to *Amam* phase, computed using DFT. Solid markers denote bulk; hollow markers indicate an oxygen vacancy. Panels (c) and (d) present the corresponding relative energies obtained from DMC calculations at two pressures (12 and 20 GPa). The labels shown in (a) and (b) are used consistently in (c) and (d) panels, respectively. A more pronounced distinction between the phases is presented in Supporting Information Figure S3.

Panels (a) and (b) in Figure 4 show the PBE+*U* relative enthalpies for bulk (La_3_Ni_2_O_7_) and vacancy phases (La_3_Ni_2_O_7−δ_), respectively. The PBE+*U* calculations show results for pressures ranging from 0 to 20 GPa. Panels (c) and (d) show the DMC-computed relative enthalpies at 12 and 20 GPa for the bulk and vacancy phases, respectively. Due to their high computational cost, we only show DMC values at these two pressures. However, the DMC calculations at these two values are sufficient to reveal the critical pressure dependence of the relative enthalpies, as discussed below. The individual absolute enthalpies for each configuration at different pressures are provided within Supporting Information Tables S6–S7.

In the PBE+*U* results, the bulk *Fmmm* and *I*4/*mmm* phases exhibit a monotonic decrease in enthalpy differences compared to the *Amam* phase with increasing pressure. The *I*4/*mmm* becomes the lowest enthalpy phase around 15 GPa. However, the *Fmmm* enthalpy is slightly higher, ranging from 6 meV/f.u. to 15 meV/f.u. throughout the pressure window. This PBE+*U* result differs with experimentally reported ordering. The *Fmmm* phase is claimed to stabilize before *I*4/*mmm*.^29,50–52^ Interestingly, we observe that, when vacancy-containing structures are considered, the *Fmmm* vacancy phase becomes favorable near 9 GPa, in better agreement with the experimentally reported sequence. This change in the relative enthalpy difference resulting from the presence of oxygen vacancies leads to decrease in the transition pressure from *Amam* to *Fmmm* phase in PBE+*U* from 18 to 9 GPa. At the same time, the stability of *I*4/*mmm* phase seems to remain largely insensitive to the presence of vacancies. Stoichiometric and vacancy-containing cases in the *Fmmm* phase both follow a similar trend.

Our many-body DMC results show at 12 GPa that the enthalpy difference between the bulk *Amam*, *Fmmm* and *I*4/*mmm* phases is within statistical uncertainty, indicating that a phase transition from *Amam* to *Fmmm*, or *I*4/*mmm* occurs. However, at 20 GPa, *Fmmm* and *I*4/*mmm* both lie well below the *Amam* phase but show relative enthalpies within error bars. The *Fmmm*-to-*I*4/*mmm* phase transition appears to occur near 19 GPa, in agreement with literature.^29^ However, we interpret this transition point with caution. The DMC error bars are too large to fully resolve the small energy difference between the *Fmmm* and *I*4/*mmm* phases at 19 GPa.

For La_3_Ni_2_O_7−δ_ at 12 GPa, DMC predicts *Fmmm* to be slightly lower in enthalpy than *Amam*, while *I*4/*mmm* phase lies slightly above the horizontal line. Furthermore, *Amam* and *I*4/*mmm* exhibit comparable relative enthalpies at both 12 and 20 GPa. In the bulk, the enthalpy of *I*4/*mmm* phase drops below the enthalpy of *Amam* near 13 GPa. By contrast, the La_3_Ni_2_O_7−δ_ counterpart remains nearly degenerate with *Amam*. For *Fmmm*-phase, bulk and vacancy cases both stabilize at 12 GPa. Importantly, when the pressure increases to 20 GPa, the relative enthalpy of the *Fmmm* phase decreases by more than 1 eV. The vacancy-bearing *Fmmm* structure drops even more steeply than the stoichiometric one. As a result, the oxygen vacancy strongly stabilizes the *Fmmm* phase, and this stabilizing effect increases with pressure.

Both DMC and PBE+*U* consistently show that introducing vacancies changes the enthalpy difference. At 20 GPa, the vacancy significantly stabilizes the *Fmmm* phase by lowering its enthalpy well below that of *Amam*. For *I*4/*mmm*, however, both methods show the opposite trend: the vacancy increases the enthalpy and therefore destabilizes the phase at 20 GPa. Otherwise, DMC and PBE+*U* predict the same qualitative vacancy effects, with DMC amplifying their magnitude.

The *Cmmm* phase does not emerge as a competing structure within the 0–20 GPa range with the *Amam* phase. However, it also shows a vacancy-induced shift in enthalpy computed using both methods. This behavior again highlights the vacancy sensitivity of the 1313-stacked *Cmmm* phase, similar to that observed in other phases with 2222 stacking.

Taken together, our PBE+*U* and DMC results show that oxygen vacancies substantially reshape the pressure-dependent stability of La_3_Ni_2_O_7_ phases. Rather than acting as a minor perturbation, oxygen deficiency emerges as a key factor, strongly affecting the phase transition under pressure. The pronounced vacancy sensitivity revealed here suggests a natural explanation for the persistent discrepancies in the transition pressures reported experimentally.^29,51,52^ These observations suggest that other intrinsic defects might influence the high-pressure behavior of La_3_Ni_2_O_7_ systems.

### 3.5 Comparison between DMC and PBE+*U*

This subsection compares PBE+*U* against benchmark many-body DMC calculations. Accordingly, we evaluated the vacancy-formation energies with both approaches across all inequivalent oxygen sites in the four La_3_Ni_2_O_7_ phases.

Figure 5 presents a scatter plot of the vacancy formation energies (FE_VO_) obtained from both methods. The associated numerical data are provided in the Supporting Information Tables S4 and S5. A summary of the lowest energy sites for oxygen vacancies is given in Table 2. Points on the dashed red *y* = *x* line would be in perfect agreement. The data show a spread of approximately 1.0 eV around this line. This variation indicates significant quantitative differences between PBE+*U* and DMC. Nevertheless, the remaining quantitative differences do not affect the main conclusions presented in Sections 3.3 and 3.4.

**Figure 5:**
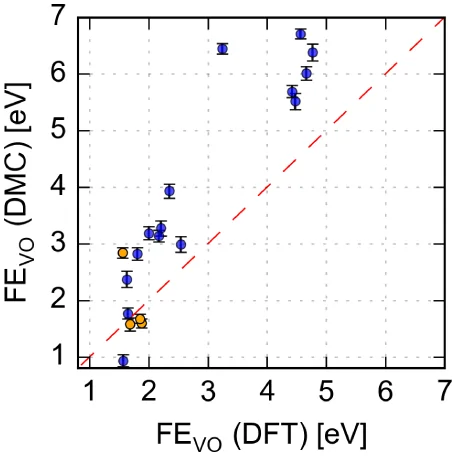
Comparison between PBE+*U* and DMC. Scatter plot comparing oxygen vacancy formation energies computed using PBE+*U* and DMC across multiple defect configurations. The dashed red line denotes the *y* = *x* reference, indicating perfect agreement between the two methods. The data points in this plot correspond to formation energies computed at 12 GPa (orange-colored circles) and 20 GPa (blue-colored circles), as listed in Table 2, and are also provided in Tables S4 and S5 of the Supporting Information.

Frequently, DMC yields higher FE_VO_ than PBE+*U*. Although PBE+*U* reproduces similar relative trends, its absolute accuracy is poor. The root mean squared deviation is 1.3 V, and the standard *R* ^2^ score is only 0.51 compared with the DMC baseline. Thus, while PBE+*U* reproduces some qualitative trends, it lacks the quantitative accuracy of DMC. Although PBE+*U* retains, roughly, the ordering of formation enthalpies found in DMC, PBE+*U* values are generally higher than DMC for all vacancies except the lowest energy one: the V1 vacancy in the *Fmmm* phase, where PBE+*U* is significantly higher. In addition, PBE+*U* has a significantly weaker FE_VO_ pressure dependence. This results in a much larger contribution of the oxygen vacancy to the stabilization of the *Fmmm* phase in DMC than in PBE+*U*. DMC is a higher-level theory that does not require tunable external parameters. The comparison with PBE+*U* demonstrates that DMC is particularly useful for understanding a material like La_3_Ni_2_O_7_ at extreme conditions.

To assess the sensitivity to the underlying DFT implementation, we compared oxygen-vacancy formation energies obtained with VASP and QE for three representative vacancy configurations in the *Amam* phase at 20 GPa. The pseudopotentials used in QE for Ni and La are significantly harder than the ones used in VASP since they include more electrons in the valence, are norm conserving, and require significantly larger energy cutoffs. However, PBE+*U* in both approaches reproduces the same qualitative ordering of vacancy stability, although QE gives slightly smaller absolute values. The detailed comparison is provided in Table S8 of the Supporting Information.

While one of the many density functional approximations available in the literature could potentially perform better than PBE+*U* for the specific case of La_3_Ni_2_O_7_, and better approximations may be developed in the future, identifying the optimal functional lies beyond the scope of this work. However, we present our data to facilitate such comparisons and support future improvements to DFT.

## 4 Discussion

Oxygen vacancies in La_3_Ni_2_O_7_ are receiving increasing theoretical attention.^6,7,9,11^ PBE+*U* studies using GGA approximation identify the apical and interbilayer oxygen sites as energetically favored for vacancy formation in La_3_Ni_2_O_7_ structures. They also show that vacancies can substantially modify Ni-*e*_*g*_ orbital occupancy and interlayer hopping, often suppressing metallization or pairing strength in the proposed high-pressure phases.^7,11^ Recent work further suggests that vacancies may order into one-dimensional chains, producing measurable optical signatures and altering intrabilayer couplings.^9^ Those results suggest that vacancy ordering, not just concentration, may play a key role. However, vacancy energetics and pressure-dependent stabilization across the competing phases *Amam*, *Fmmm*, *I*4/*mmm*, and *Cmmm* remain poorly characterized. Existing studies rely mostly on PBE+*U* approximations and focus on only one low-pressure phase and a single high-pressure phase.^4,34^ Therefore, a comprehensive set of calculations, spanning both bulk and defect structures across increasing pressure, is essential for understanding La_3_Ni_2_O_7_.

In this work, we provide a key accurate many-body benchmark of mean-field predictions^9,58^ to address the lack of experimental information at high pressure commonly used to adjust DFT approximations.

In our many-body DMC calculations, the inner apical oxygen sites emerge as the most favorable vacancy positions for all phases considered. This trend is consistent with experimental observations and agrees qualitatively with prior DFT studies on layered nickelates and related Ruddlesden–Popper phases.^6,7,9^ These inner apical sites are in a distinct structural environment between adjacent NiO_6_ octahedral layers, where the local bonding is highly anisotropic. The weaker electrostatic potential and reduced covalency at these positions reduce the energy cost of removing an oxygen atom compared with the more strongly bonded equatorial sites.^6,7^ In addition, the inner apical environment offers greater lattice flexibility, enabling local relaxation around the vacancy with minimal strain.^8^ Together, these electronic and elastic factors make inner apical vacancies energetically preferred. Their formation can therefore drive structural rearrangements and stabilize specific phases under high pressure.^8,59^

Furthermore, our DMC results indicate that, without vacancies, phase transitions from *Amam* to *Fmmm* and from *Fmmm* to *I*4/*mmm* occur near 10 and 17 GPa, respectively. Although the DMC error bars are too large to be certain and pinpoint the exact transition pressures. By contrast, a phase transition from *Amam* to *I*4/*mmm* occurs in PBE+*U* at 16 GPa, while the *Fmmm* phase remains higher in energy in the range of pressures studied. For the structures with oxygen vacancies, with DMC, the *Amam*-to-*Fmmm* and *Fmmm*-to-*I*4/*mmm* transitions appear at approximately 10 and 11 GPa, respectively. In this case, the uncertainty in the transition pressure is about 2 GPa, which is insufficient to resolve the precise boundary. However, the vacancy-stabilization effect in the *Fmmm* phase is unambiguous. The transition pressures for the high-pressure phases lie within the range of experimentally reported pressure variation.^29^ We also note that experimental studies disagree on phase transition pressures.^29,51,52^

We note that these results correspond to the zero-temperature enthalpy limit of the present calculations. Under experimental conditions, finite-temperature free-energy contributions like vibrational entropy may further modify the relative stability of competing phases and shift the transition pressures quantitatively. Although these effects are not explicitly included here, they are not within the scope of this work and are not expected to change the conclusion that oxygen vacancies play a significant role in the phase transition with pressure. On the other hand, the configurational entropy associated with the oxygen vacancy for the *Amam*, *I*4/*mmm*, and *Fmmm* phases should remain unchanged under experimental conditions since, in all cases, the lowest energy occurs at the inner apical site (V1), thus the number of vacancies per site is the same. Furthermore, the energy difference between the V1 sites and other sites is significantly higher, above 0.6 eV (∼7000 K) than relevant experimental temperatures (below 300 K).^52^ The *Cmmm* phase zero-temperature enthalpy of formation is too high compared to the other phases for configurational entropy or phonon entropy to make any significant difference.

## 5 Conclusion

We carried out a systematic many-body DMC investigation of oxygen vacancy energetics across the four experimentally reported phases of La_3_Ni_2_O_7_: *Amam*, *Fmmm*, *I*4/*mmm*, and *Cmmm*. Our work studies the magnetic ground states, the calculation of vacancy-formation energies, and the pressure dependence of enthalpy for each phase. Our goal is to understand the influence of oxygen stoichiometry on the phase transitions as a function of pressure.

Our DMC results provide an accurate picture of how vacancies influence both the structural and magnetic behavior of the material. All phases adopt AFM ground states. However, the *Amam* and *I*4/*mmm* structures exhibit in-plane ferromagnetism coupled antiferromagnetically along the out-of-plane direction, whereas *Cmmm* and *Fmmm* favor AFM ordering in both directions. This diversity highlights the delicate balance of competing interactions within the nickelate framework.

Our analysis of vacancy formation across all symmetry-inequivalent oxygen sites shows a strong site preference. Inner apical oxygens are the most favorable sites for vacancy formation in the *Amam*, *Fmmm*, and *I*4/*mmm* phases, while the inner planar oxygen in *Cmmm* has the lowest vacancy formation energy. By contrast, the outer apical sites consistently show the highest formation energies in every phase. These site-dependent trends are in qualitative agreement with recent theoretical calculations with approximated PBE+*U* functionals^9^ and experiments.^6^

The analysis of how the enthalpy of different phases responds to pressure reveals a strong sensitivity to the presence of oxygen vacancies. We calculated the relative enthalpy of high-pressure phases (*Fmmm*, *I*4/*mmm*, and *Cmmm*) with respect to the low-pressure *Amam* phase. Vacancy-containing structures show substantially different relative enthalpies compared to their stoichiometric counterparts. The magnitude of this effect increases with pressure. For example, the *Fmmm* vacancy-containing phase at 20 GPa is 1.18(15) eV lower in relative enthalpy than that at 12 GPa. The stoichiometric *Fmmm* phase also shows a reduction in the relative enthalpy with pressure, but the effect is considerably smaller, 0.37(11) eV. Similar vacancy-driven shifts in relative enthalpy appear in the other phases, with varying strengths and directions. These trends demonstrate that oxygen vacancies, at concentrations comparable to experimental ones, are not minor perturbations but key contributors to the high-pressure behavior of La_3_Ni_2_O_7_.

Taken together, the results uncover the role of oxygen vacancies as a driving force for structural transitions, helping to reconcile discrepancies between experimental observations reported for different samples. These findings suggest that other intrinsic defects may have a similar influence on phase stability. The high-accuracy DMC framework is critically important to study materials at extreme conditions, where experimental information commonly used to fit PBE+*U* approximations is difficult to obtain. Our DMC results provide reliable insights into these defect and phase energetics, including correlation-driven effects and those arising from defect chemistry. These insights can guide future high-pressure experiments, clarify the conditions under which different structural phases emerge, and inform ongoing discussions of phase stability and electronic behavior in layered nickelates.

## Supplementary Material



## Data Availability

The data used to generate all of the figures and tables presented in this manuscript have been deposited in the Oak Ridge National Laboratory Constellation repository and will be accessible at the DOI: 10.13139/OLCF/3382294.

## Notes

AFM spin configurations; calculation of Heisenberg exchange parameters; pressure-dependent vacancy configurations; oxygen vacancy formation energy data; pressure-dependent enthalpy; comparison between DFT methods; Hubbard *U* comparison; DMC time step test (PDF)
