# Supplementary material for: The Role of Intrinsic Defects in the Structural Transition of Superconducting La3Ni2O7: A Many-Body DMC Prediction
Source: Chem Mater. 2026 Sep 4;38(18):9450–60. doi: 10.1021/acs.chemmater.6c01607 (PMC13602844; doi:10.1021/acs.chemmater.6c01607)
Supplement: Supplementary file 1 [file cm6c01607_si_001.pdf]

Supporting Information for Publication

The role of intrinsic defects in the structural transition of superconducting $La_3Ni_2O_7$: A many-body DMC prediction

Abdul Ghaffar*[1], Kayahan Saritas*[1], and Fernando A. Reboredo*[1]

[1]Materials Science and Technology Division, Oak Ridge National Laboratory, Oak Ridge, Tennessee 37831, USA

1 AFM Spin Configurations

*Corresponding author1: abdulg@ornl.gov
*Corresponding author2: saritask@ornl.gov
*Corresponding author3: reboredofa@ornl.gov

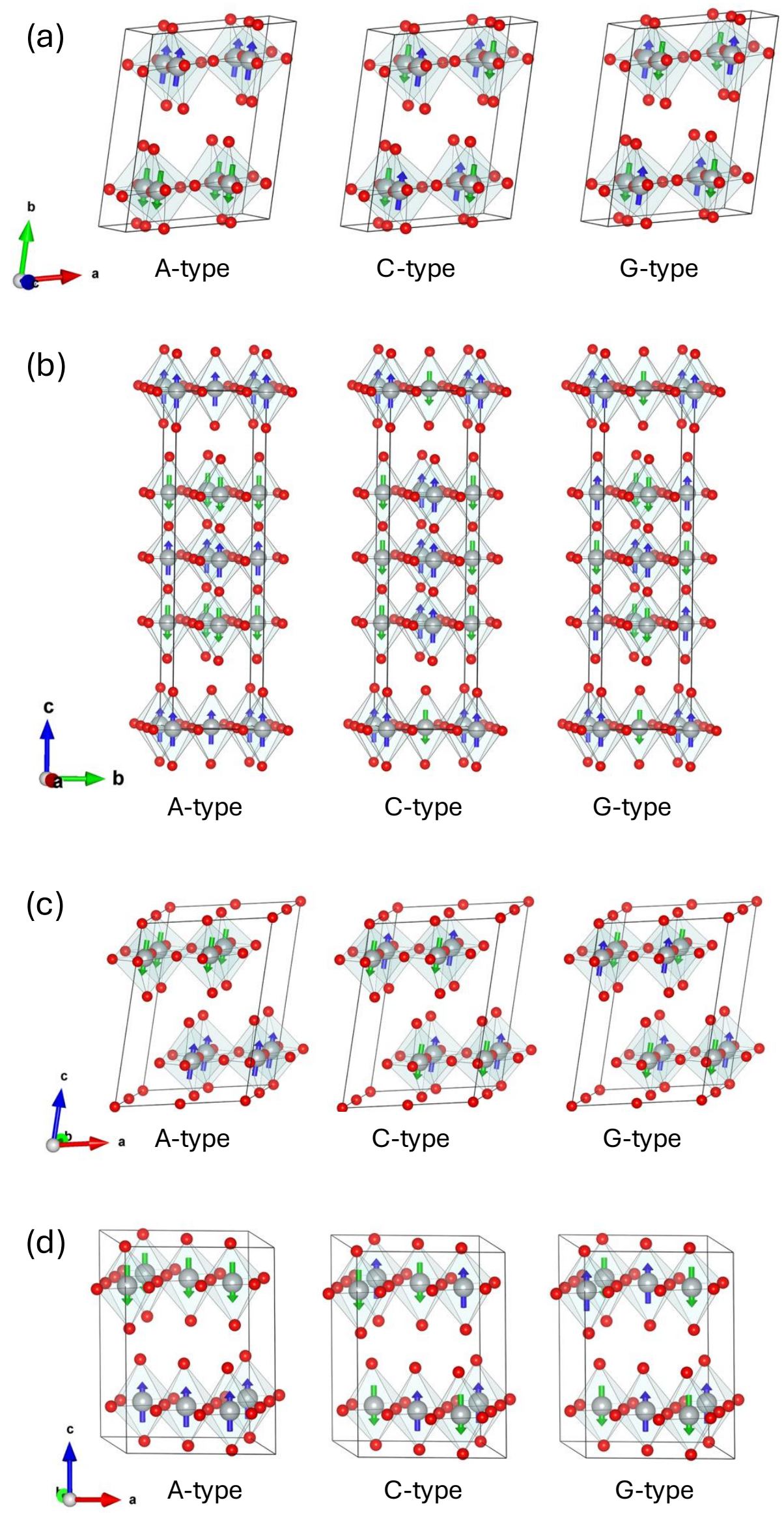

Figure S1: **Spin orientations of different AFM configurations.** Three antiferromagnetic configurations: A-type, C-type, and G-type. The spin ordering is shown for all the four crystal phases within 4-formula-unit supercells: (a) $Amam$, (b) $Cmmm$, (c) $I4/mmm$, and (d) $Fmmm$. Up- and down-spin orientations are indicated by blue and green arrows, respectively. Silver and red spheres denote Ni and O atoms. La atoms are omitted for clarity to highlight the magnetic and oxygen coordination environments.

Table S1: Energies of the phases in different magnetic configurations (4fu bulk supercell) computed using QMC (DMC) method.

Systems	Energy (eV)	Error (eV)
Amam (FM)	-59433.832443	0.093879
Cmmm (FM)	-59432.694319	0.056355
Fmmm (FM)	-59434.575551	0.048055
*I*4/*mmm* (FM)	-59434.460526	0.060382
Amam (A-type)	-59434.904400	0.058831
Cmmm (A-type)	-59430.496205	0.063729
Fmmm (A-type)	-59435.119028	0.061416
*I*4/*mmm* (A-type)	-59435.433037	0.055484
Amam (C-type)	-59434.550821	0.084546
Cmmm (C-type)	-59433.616782	0.076573
Fmmm (C-type)	-59435.356450	0.056817
*I*4/*mmm* (C-type)	-59435.203433	0.061253
Amam (G-type)	-59433.016682	0.091131
Cmmm (G-type)	-59430.721662	0.096437
Fmmm (G-type)	-59434.617632	0.147486
*I*4/*mmm* (G-type)	-59434.048636	0.051892

Table S2: Energies of the phases in different magnetic configurations (4fu bulk supercell) computed using DFT (PBE+U) method.

Systems	Energy (eV)
Amam (FM)	-286.033565
Cmmm (FM)	-285.801760
Fmmm (FM)	-286.153720
*I*4/*mmm* (FM)	-285.929440
Amam (A-type)	-286.499247
Cmmm (A-type)	-285.707584
Fmmm (A-type)	-286.371867
*I*4/*mmm* (A-type)	-286.315807
Amam (C-type)	-286.214612
Cmmm (C-type)	-285.969493
Fmmm (C-type)	-286.530703
*I*4/*mmm* (C-type)	-286.375530
Amam (G-type)	-285.812545
Cmmm (G-type)	-286.068790
Fmmm (G-type)	-286.069667
*I*4/*mmm* (G-type)	-285.938500

2 Extraction of Heisenberg Exchange Parameters

Table S3: Comparison of exchange parameters, intralayer coupling ($J_{\parallel}$) and interlayer coupling ($J_{\perp}$) from PBE+U and DMC, on the bulk phases at 20 GPa. The units for J are meV per Ni–Ni pair.

**Phase**	**PBE+U**		**DMC**	
	$J_{\parallel}$	$J_{\perp}$	$J_{\parallel}$	$J_{\perp}$
$Amam$	7.02	-24.10	16.24 ± 1.91	-72.39 ± 3.82
$Cmmm$	-14.69	-0.14	-31.89 ± 8.35	141.48 ± 3.85
$I4/mmm$	-0.72	-22.40	10.01 ± 1.22	-57.30 ± 2.54
$Fmmm$	-1.04	-22.24	-3.88 ± 2.36	-38.33 ± 6.64

Magnetic exchange couplings were obtained by mapping first-principles total energies of several collinear spin configurations onto a Heisenberg model,

$$H = -\sum_{i<j} J_{ij}\, \mathbf{S}_i \cdot \mathbf{S}_j. \tag{1}$$

For collinear spins, $\mathbf{S}_i \cdot \mathbf{S}_j = S^2 \sigma_i \sigma_j$ with $\sigma_i = \pm 1$. In the structural input files, magnetic sites were identified by labels `Ni1` and `Ni2`, and we assigned $\sigma_i = +1$ for `Ni1` and $\sigma_i = -1$ for `Ni2` (all other atoms have $\sigma_i = 0$). A fixed spin length $S = 1.5$ was used.

Couplings were parameterized by distance shells to include up to 3 shells, including first, second, and third-nearest neighbors (NN). In $La_3Ni_2O_7$, the first, and the second NN represent the intralayer and interlayer Ni–Ni pairs, respectively. For configuration α, we computed the shell-resolved spin-correlation sums

$$C_n^{(\alpha)} = \sum_{(i<j)\in n} \sigma_i^{(\alpha)} \sigma_j^{(\alpha)}. \tag{2}$$

The total energy then reads

$$E_\alpha = E_0 - S^2 \sum_n J_n\, C_n^{(\alpha)}. \tag{3}$$

Subtracting the FM energy eliminates E_0 and yields linear equations for the shell couplings J_n:

$$\Delta E_\alpha \equiv E_\alpha - E_{\mathrm{FM}} = -S^2 \sum_n J_n \left(C_n^{(\alpha)} - C_n^{(\mathrm{FM})} \right). \tag{4}$$

We built this system using all antiferromagnetic (AFM) configurations with zero net collinear magnetization on the magnetic sublattice, and solved for $\{J_n\}$ by linear least squares.

From the data in Tables S2 and S1, we computed the corresponding J values by mapping the energies of different magnetic configurations onto a Heisenberg model as given in the Table S3. From the DMC results, we find a clear trend in J. In every phase, $J_\perp$ is much larger than $J_\parallel$. For example, in the *Amam* phase, $J_\perp$ is strongly negative (-72.39 ± 3.82 meV). This indicates stable antiferromagnetic interlayer alignment. In contrast, $J_\parallel$ remains much smaller and positive 16.24 ± 1.91, suggesting intralayer FM coupling. The same order exists in *Cmmm*, *I*4/*mmm*, and *Fmmm*. The sign and magnitude of individual couplings vary with structure.

PBE+U reproduces the same qualitative trend for *Amam*, *I*4/*mmm*, and *Fmmm*. However, for *Cmmm* it predicts $|J_\parallel|$ to be significantly larger than $|J_\perp|$. In contrast, DMC yields larger $|J|$ values and consistently identifies $|J_\perp|$ as dominant across all phases. The discrepancy in the *Cmmm* phase is also evident in Tables S2 and S1, where PBE+U predicts G-type ordering, whereas DMC favors C-type ordering. In *Amam*, PBE+U gives a more negative $J_\perp$ (-24.10 meV) and a smaller $J_\parallel$ (7.02 meV), similar to DMC. However, in *Cmmm* and *I*4/*mmm*, the signs of J differ between DMC and PBE+U.

The overall stronger interlayer magnetic coupling in $La_3Ni_2O_7$ is corroborated by experiments [1].

3 Pressure-dependent vacancy configurations

To explore pressure-dependent energetics of the $La_3Ni_2O_7$ vacancy phase further around the sharp discontinuity in the enthalpy landscape between 12 and 13.5 GPa in Figure S2, we re-calculated the trend in two different pressure sweeps; forward sweep from 12 to 20 GPa, and backward sweep from 13.5 to 0 GPa given in SI Figure S2. When pressure is increased from ambient conditions, the system undergoes an abrupt stabilization of the vacancy-containing *Fmmm* configuration relative to the *Amam* phase, followed by a smooth evolution up to 20 GPa. In contrast, the reverse pressure sweep shows no corresponding discontinuity, instead exhibiting a continuous trajectory that bypasses the transition point. This pronounced hysteresis suggests a finite energy barrier separating the two structural basins, highlighting how oxygen vacancies modify the phase boundary and stabilize metastable configurations under compression.

4 Oxygen vacancy formation energy data

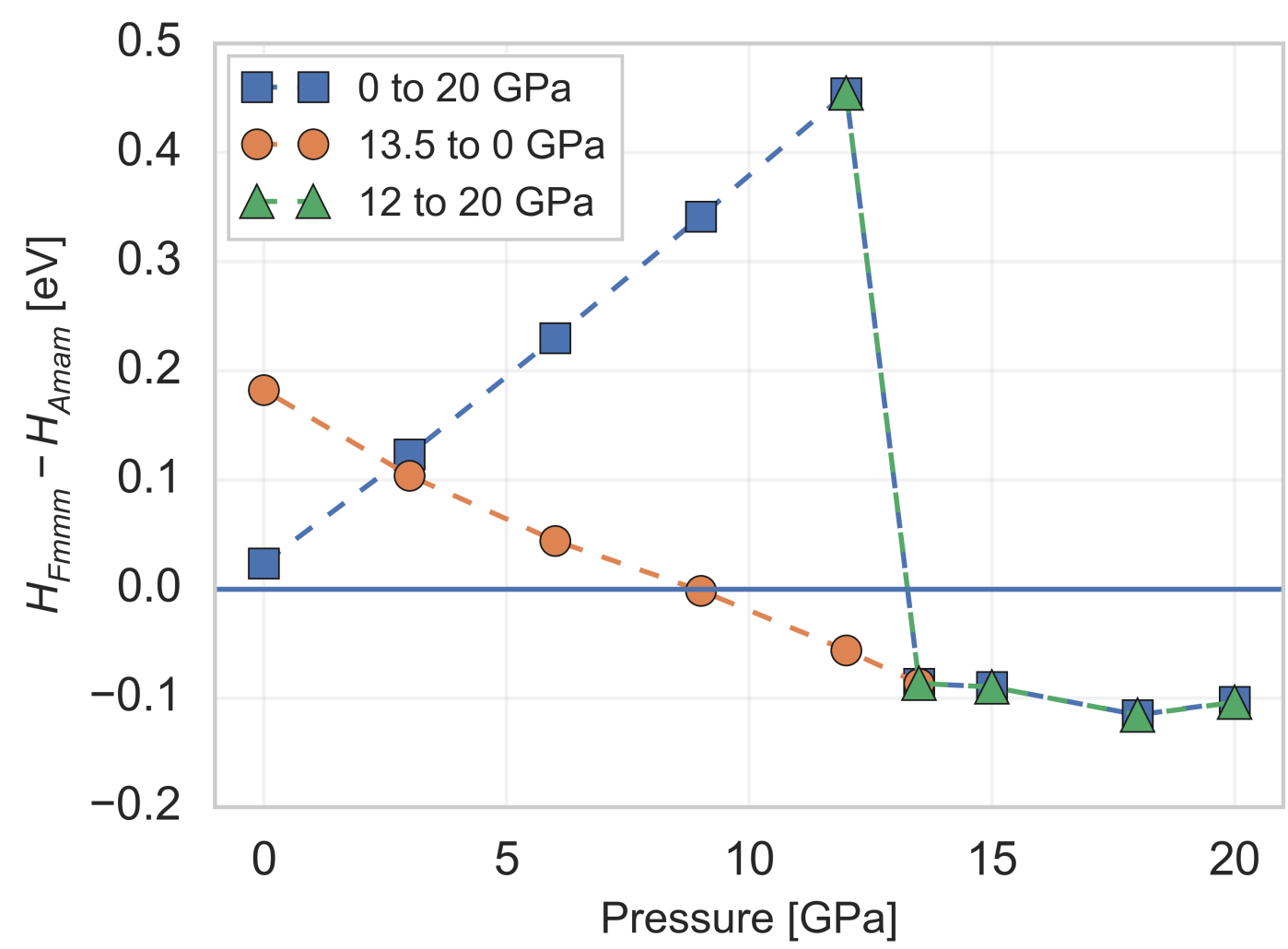

Figure S2: **Relative energy of the *Fmmm* vacancy configuration.** Pressure-dependent relative energy of the *Fmmm* vacancy configuration compared to the *Amam* phase. Three curves are shown: one spanning the full range from 0 to 20 GPa, and two additional sequences centered around the transition region, covering 12–20 GPa and 13.5–0 GPa, respectively.

Table S4: Vacancy formation energies grouped by phases computed using DMC method at 20 GPa.

**Vacancy**	**FE$_{\mathbf{VO}}$ (eV)**	**Error (eV)**
Amam		
V2	2.988206	0.138489
V3	5.516439	0.146259
V1	1.769592	0.098957
Cmmm		
V3'	6.380994	0.149503
V2'	6.447715	0.095602
V3“''	6.010784	0.117968
V5	3.272981	0.136382
V1'	3.186631	0.117721
V4	2.822940	0.107772
I4/mmm		
V2	3.931562	0.122654
V3	6.711511	0.090337
V1	2.370002	0.142699
Fmmm		
V2	3.140461	0.102086
V3	5.691249	0.107638
V1	0.932098	0.106662

Table S5: Vacancy formation energies grouped by phases computed using DFT method at 20 GPa.

**Vacancy**	$\mathbf{FE_{VO}}$ **(eV)**
Amam	
V2	2.536940
V3	4.478505
V1	1.647972
Cmmm	
V3'	4.772212
V2'	3.242493
V3“'	4.658876
V5	2.204115
V1'	2.002656
V4	1.835172
I4/mmm	
V2	2.346083
V3	4.566465
V1	1.676000
Fmmm	
V2	2.167499
V3	4.425889
V1	1.568362

5 Pressure-dependent Enthalpy

Table S6: DFT (PBE+U)-calculated enthalpies for the *Amam* and *Fmmm* configurations, shown for both bulk and vacancy (Vac.) structures across a range of pressures. Enthalpies are reported in eV per 4-fu cell of $La_3Ni_2O_7$. The vacancy structures in both *Amam* and *Fmmm* phases correspond to the V1 site, defined as the inner apical oxygen position.

Pressure (GPa)	Bulk *Amam*	Vac. *Amam*	Bulk *Fmmm*	Vac. *Fmmm*
0.0	-357.306120	-350.190500	-356.649760	-350.166830
3.0	-346.128460	-339.074150	-345.640440	-338.970210
6.0	-335.167010	-328.183290	-334.817350	-328.139010
9.0	-324.398530	-317.492590	-324.163670	-317.494090
12.0	-313.808830	-306.984640	-313.667070	-307.040780
13.5	-308.586070	-301.803770	-308.473750	-301.890460
15.0	-303.383620	-296.643440	-303.316050	-296.733150
18.0	-293.109120	-286.452360	-293.100510	-286.567770
20.0	-286.338840	-279.750000	-286.372310	-279.851780

Table S7: DFT (PBE+U)-calculated enthalpies for the *Cmmm* and $I4/mmm$ configurations, shown for both bulk and vacancy (Vac.) structures across a range of pressures. Enthalpies are given in eV per 4-fu cell of $La_3Ni_2O_7$. The vacancy configurations in the *Cmmm* and $I4/mmm$ phases correspond to the inner planar (V4) and inner apical (V1) sites, respectively.

Pressure (GPa)	Bulk *Cmmm*	Vac. *Cmmm*	Bulk $I4/mmm$	Vac. $I4/mmm$
0.0	-356.100520	-349.938010	-356.709690	-349.537340
3.0	-345.098130	-338.768660	-345.694760	-338.743320
6.0	-334.258180	-327.912090	-334.868780	-327.965220
9.0	-323.569560	-317.145160	-324.211070	-317.360300
12.0	-313.059260	-306.554760	-313.711620	-306.914700
13.5	-307.868050	-301.261250	-308.517400	-301.756750
15.0	-302.721020	-296.059630	-303.357430	-296.617450
18.0	-292.563460	-285.783800	-293.139640	-286.456080
20.0	-285.808910	-279.028290	-286.398140	-279.754590

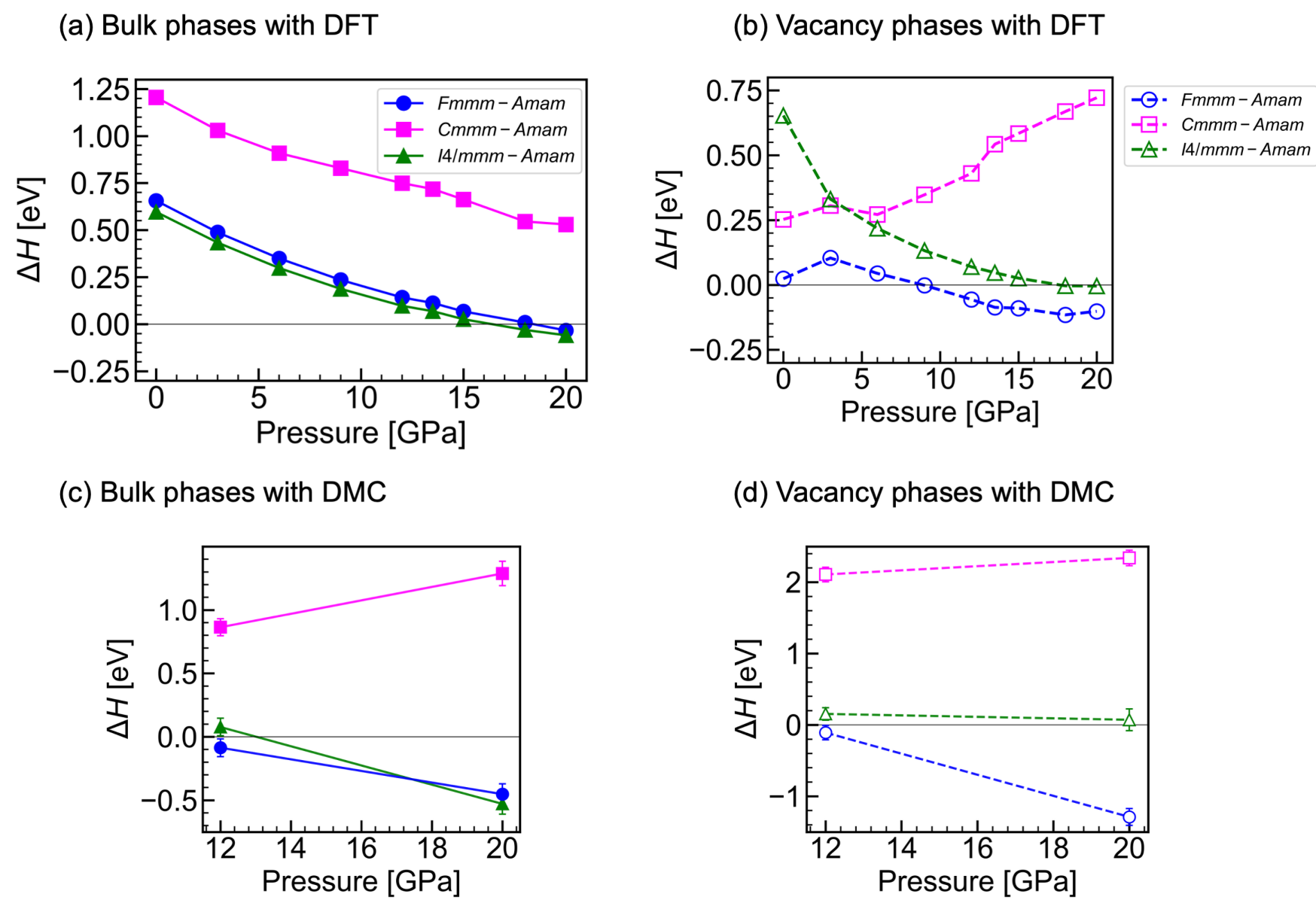

Figure S3: **Relative enthalpy of high-pressure phases.** Panel (a) and (b) show the pressure-dependent enthalpy of the three high-pressure phases ($Cmmm$, $I4/mmm$, and $Fmmm$) relative to $Amam$ phase, computed using DFT. Solid markers denote bulk; hollow markers indicate an oxygen vacancy.; Panels (c)-(d) present the corresponding relative energies obtained from DMC calculations at two pressures (12 GPa and 20 GPa). The labels shown in (a), and (b) are used consistently in both (c), and (d) panels, respectively. The slight upward kink in panel (b) for $Fmmm$ phase can be attributed to the behavior shown in Figure S2.

6 Comparison between VASP and QE

Table S8: Comparison of oxygen-vacancy formation energies (in eV) obtained with VASP and Quantum ESPRESSO (QE) for three representative vacancy configurations in the *Amam* phase at 20 GPa.

Configuration	QE	VASP
V_1 (inner apical)	1.4334	1.6480
V_2 (planar)	2.0593	2.3716
V_3 (outer apical)	3.9453	4.4785

7 Hubbard U: 3.5 eV vs 4.5 eV

Table S9: PBE+U total energies (in eV per formula unit, or as defined in the main text) for the *Fmmm* (20 GPa) phase with and without an oxygen vacancy, computed using $U = 3.5$ and 4.5 eV. The resulting vacancy formation energies (FE) differ by only ~ 18 meV.

System	$U = 3.5$ eV	$U = 4.5$ eV
Fmmm (pristine)	-286.37231	-283.88600
Fmmm (O-vacancy)	-279.85178	-277.38337
FE	1.5751	1.5572
ΔFE $\approx$ 18 meV		

8 DMC time step test

Table S10: Representative DMC time-step test for the *Fmmm* phase (20 GPa). Total energies are given in Ha.

System	$\tau = 0.02$ Ha^{-1}	$\tau = 0.01$ Ha^{-1}
Pristine	$-2186.753823 \pm 0.005286$	$-2186.666282 \pm 0.003241$
Vacancy	$-2170.715540 \pm 0.005898$	$-2170.625027 \pm 0.004353$
ΔE	16.038283 ± 0.00792	16.041255 ± 0.0054

References

[1] Tao Xie, Mengwu Huo, Xiaosheng Ni, Feiran Shen, Xing Huang, Hualei Sun, Helen C. Walker, Devashibhai Adroja, Dehong Yu, Bing Shen, Lunhua

He, Kun Cao, and Meng Wang. Strong interlayer magnetic exchange coupling in $la_3ni_2o_7$ revealed by inelastic neutron scattering. *Science Bulletin*, 69(20):3221–3227, October 2024.
